# Predicting Survival Outcomes for Patients with Ovarian Cancer Using National Cancer Registry Data from Taiwan: A Retrospective Cohort Study

**DOI:** 10.1089/whr.2024.0166

**Published:** 2025-01-21

**Authors:** Amrita Chattopadhyay, Ya-Ting Wu, Han-Ching Chan, Yi-Ting Kang, Ying-Cheng Chiang, Chun-Ju Chiang, Wen-Chung Lee, Tzu-Pin Lu

**Affiliations:** ^1^ Institute of Epidemiology and Preventive Medicine, Department of Public Health, College of Public Health, National Taiwan University, Taipei, Taiwan.; ^2^ Department of Obstetrics and Gynecology, College of Medicine, National Taiwan University, Taipei, Taiwan.; ^3^ Department of Obstetrics and Gynecology, National Taiwan University Hospital, Taipei, Taiwan.; ^4^ Department of Obstetrics and Gynecology, National Taiwan University Hospital Hsin-Chu Branch, Hsinchu, Taiwan.; ^5^ Taiwan Cancer Registry, Taipei, Taiwan.; ^6^ Department of Public Health, Institute of Health Data Analytics and Statistics, College of Public Health, National Taiwan University, Taipei, Taiwan.; ^7^ Population Health Research Center, National Taiwan University.

**Keywords:** Epithelial ovarian cancer, Taiwan Cancer Registry (TCR), Surveillance Epidemiology and End Results registry (SEER), survival prediction, racial differences

## Abstract

**Background:**

Ovarian cancer is one of the top seven causes of cancer deaths. Incidence of ovarian cancer varies by ethnicity, where Asian women demonstrate lower incidence rates than non-Hispanic Blacks and Whites. Survival prediction models for ovarian cancer have been developed for Caucasians and Black populations using national databases; however, whether these models work for Asians is unclear. Therefore, a retrospective cohort study was conducted to develop survival prediction models for patients with epithelial ovarian cancer from a Taiwan Cancer Registry (TCR) who underwent de-bulking and chemotherapy, with the aim to identify variables that can predict prognosis accurately. Patients diagnosed with OC from TCR were included.

**Method:**

Two prognostic models (M1 and M2) were developed: M1 utilized clinical variables only, M2 additionally included cancer-specific variables with the aim to improve the accuracy. All methods were repeated independently for patients with only serous ovarian cancer. All findings for model M1 were validated among Black, White, and Asian populations from Surveillance, Epidemiology, and End Results (SEER) database and 10-fold internal cross-validations. Due to absence of cancer-specific site variables in SEER, model M2 was only internally validated. Cox-proportional hazards regression analysis was performed and a stepwise strategy with Akaike-information criterion was used to select appropriate variables as predictors to develop both M1 and M2.

**Results:**

The c-index values of both models were >0.7 in both TCR and SEER populations for epithelial ovarian cancer. Calibration analysis demonstrated good prediction performance with the proportional difference between predicted and observed survival to be <5%. The performance was similar for the subset of patients with serous epithelial ovarian cancer. Notably, no significant racial differences were observed.

**Conclusion:**

The prognostic models proposed in this study can potentially be used for identifying patients, especially from Taiwan, at higher risk of ovarian cancer mortality early on, leading to improved prognosis, through shared decision-making between physicians and patients.

## Introduction

Ovarian cancer is one of the most commonly occurring cancers in women and is one of the seven leading causes of death due to cancer, not only in Taiwan but worldwide.^1^ A lack of specific symptoms and effective biomarkers for early detection often leads to a late diagnosis, which is the primary factor contributing to a poor prognosis in ovarian cancer.^2^ Incidence of ovarian cancer varies by ethnicity, where Asians demonstrate lower incidence rates as opposed to non-Hispanic blacks and non-Hispanic whites.^3^ As of 2018, the age-standardized incidence and death rates of ovarian cancer have risen from 7.7 to 9.68 and from 2.86 to 3.42, respectively, per 100,000 people in Taiwan^4,5^ and are 6.6 and 4.2 per 100,000 population, globally.^6^ Furthermore, prevalence of the risk factors associated with ovarian cancer differ by ethnicity and histology, and not much is understood about the underlying etiology of it.^1^ Evidence strongly indicates the existence of racial differences in the incidence of ovarian cancer and that survival is better in Asians while worse in Blacks.^7–9^ Survival prediction models for ovarian cancer have been developed for Caucasians and Black populations using national cancer databases; however, whether these models work for Asian populations is unclear.^10,11^

Approximately 45% of ovarian cancers in Taiwan are diagnosed at stage I, while 30% are diagnosed at stage III^12,13^ Significant heterogeneity in survival rates is observed among patients diagnosed at different stages, with 5-year survival rates >80% for stage I, <50% for stage II, 40–50% for stage III, and <25% for stage IV.^7,8^ Ovarian cancer further demonstrates heterogeneous histology and can be divided into three main subtypes: Epithelial ovarian cancer (most frequent comprising of 90% of ovarian cancer), germ cell ovarian cancer and sex-cord stromal cell ovarian cancer (both accounting for ∼2–4%^5,7^ of all ovarian cancers). Furthermore, based on the cell histology, epithelial ovarian cancer is classified into serous, mucinous, endometrioid, and clear cell types, each demonstrating different 5-year survival rates.^7,8^ Improved treatments and diagnostic strategies are in use, despite which the prognosis of ovarian cancer remains poor.

Most ovarian cancers present at an advanced stage, with poor survival and high recurrence rate.^14,15^ Precise risk prediction models are a potential avenue to personalized therapy. Understanding clinical risk factors in context of Taiwanese patients will reveal significant drivers of prognosis in ovarian cancer, affecting survival in patients who underwent treatment, from Taiwan. This study, therefore, aimed to develop a clinical prediction model for patients with epithelial ovarian cancer, who underwent de-bulking and chemotherapy, by utilizing data from the Taiwan Cancer Registry (TCR).^16^ The goal is to identify variables that can accurately predict cancer specific survival (CSS) and overall survival (OS), post-surgery and -chemotherapy, in patients with ovarian epithelial cancer. This study is in line with our prior works on breast cancer and colon cancer where we have created publicly available platforms (https://preparetaiwan.org/en/prediction/colon_cancer_advance) that can predict CSS and OS for Asian patients who undergoes surgery to assist clinicians in making treatment decisions. Due to the disproportionate number of patients with epithelial ovarian cancer and the differences in pathological characteristics among the ovarian cancer types, this study focused only on epithelial ovarian cancer patients (referred to as ovarian cancer from here on).^17^ The proposed models were extensively evaluated using internal and external validation using patients of different ethnicities from the Surveillance, Epidemiology, and End Results (SEER) registry.^18^ Finally, the survival prediction models were recreated using only patients with serous ovarian cancer to avoid unknown interference from different subtypes.

## Methods

Ethical approval for this study (201910027W) was provided by the institutional review board of National Taiwan University Hospital, Taipei, Taiwan.

### Datasets

**FIG. 1.**
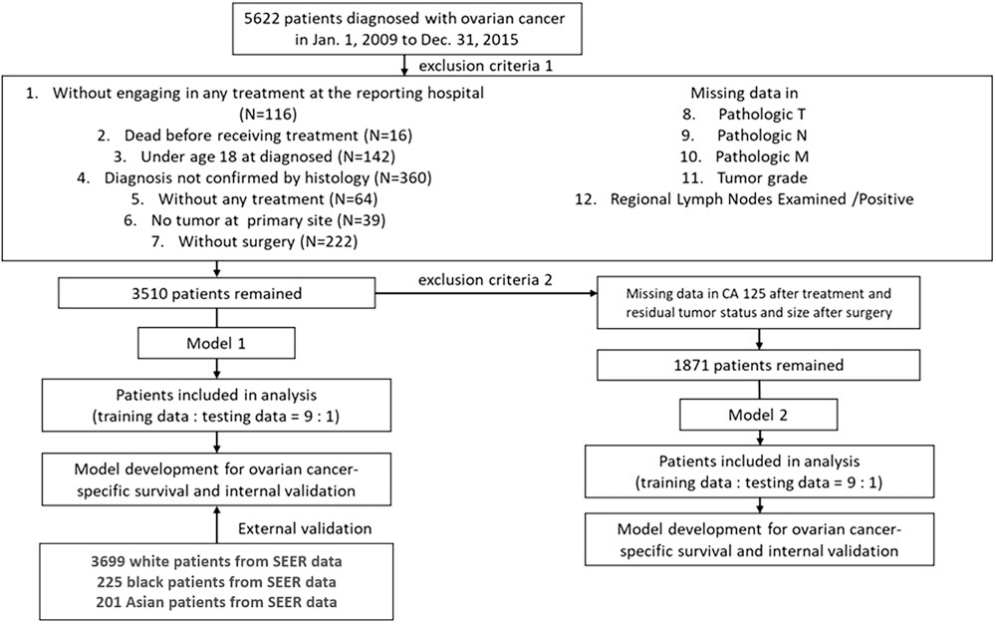
Inclusions and exclusion criteria for study subjects.

A total of 5,622 patients diagnosed with epithelial ovarian cancer between January 1, 2009, and December 31, 2015, who underwent de-bulking and chemotherapy, were identified from TCR and followed until December 31, 2017. TCR is a population-based cancer registry in Taiwan that houses information on the incidence, care, and survival of patients newly diagnosed with cancer from hospitals with more than 50 beds, collected since 1979.^16,19^ International Classification of Disease codes (http://sc-dr.tw/news/104/ICD9210/ICD-9-CM_ICD-10-CM.pdf) were used to select patients with epithelial ovarian cancer and determine subtypes (Supplementary Table S1). We conducted a retrospective cohort study with two separate analyses: first, 3,510 study subjects were included to develop prognostic model 1 (M1) where 2,112 patients were excluded based on several exclusion criteria (“exclusion criteria 1”) (Supplementary Data S1. A 9:1 ratio were used to train and test M1 *via* a 10-fold cross-validation strategy. The second prognostic model (M2) was developed using additional ovarian cancer and tumor-specific variables and a second exclusion criteria (exclusion criteria 2) (Supplementary Data S1) was used. Internal validation using 10-fold cross-validation was implemented for M2. Figure 1 summarizes all the inclusions and exclusions and gives an overall summary of the study design. External validation was conducted for model M1 with subjects from different races: White, Black, and Asian patients from the SEER database^18,20^ (Supplementary Data S1).

### Variables

Clinical, demographic, and tumor-specific variables from the long form version of TCR was utilized in this study^19^ (Supplementary Data S1). Ovarian cancer site-specific variables that were used to develop M2 were not available in SEER and therefore M2 was validated *via* cross-validation only and not by SEER.

### Study endpoints

The primary study outcome for each of the models, M1 and M2, was CSS. Additionally, prognostic models for OS were developed for patients with ovarian cancer following the same analysis procedure (details in Supplementary Data S1).

### Model development

In the first step, a univariate Cox proportional hazards regression analysis was performed on all clinico-pathological variables and *a priori* reported risk factors for ovarian cancer, to unravel the variables most likely to be associated with survival.^21^ A *p-*value <0.05 was used as the significant criterion, and the variables that reached significance were included in the multiple regression Cox proportional hazards model for the second step. Additionally, interaction effects using the variables that were significant in univariate step were also included in order to increase the explanatory power of the model.^22^ Stepwise regression was employed for conducting variable selection using Akaike information criterion (AIC) that measures the goodness-of-fit of the statistical model. As lower AIC implies a better fit, the predictors of the final prognostic model were determined by iteratively adding the variables and interaction terms until the desired minimum AIC was attained.^23^ Results were expressed as hazard ratios (HRs). Model M1 included as predictors all demographic, diagnostic, and clinical parameters which were significant in the preceding steps. For model M2, in addition to the predictors used in M1, cancer site-specific variables from TCR were utilized. The R packages “StepReg”^24^ and “survival”^25^ were utilized for conducting stepwise regression analysis and model development, respectively. A *p-*value threshold of 0.05 was set to depict significance.

### Serous epithelial ovarian carcinoma analysis

All patients with epithelial ovarian cancer from TCR were further stratified based on the cancer subtype, and only patients with serous subtype were chosen to do an independent analysis. Serous ovarian cancer has the highest incidence rate in ovarian cancer. In order to avoid the unknown interference brought by different types, only patients with serous ovarian cancer were selected to establish a survival prediction model. It was established and validated again using the SEER database.

### Model evaluation and validation

Discrimination analysis and calibration analysis were performed to evaluate the performance of all models that were proposed.^26,27^ Harrell’s c-index was calculated to determine the concordance between the predicted survival and observed survival, representing the discrimination ability of the prediction model. A c-index <0.5 implies poor model predictive ability, >0.7 implies good predictability, and >0.8 depicts robust predictive ability, while >0.9 means that the model has excellent predictive ability. Calibration analysis confirms whether the proportion of the difference between predicted and observed mortality is significant for a given follow-up time. The proportion test was utilized, and a *p-*value <0.05 was set for significance. All of the above were done *via* an internal 10-fold cross-validation strategy.

The SEER database was utilized as an external dataset to validate model M1 and determine if it can be used to predict survival for patients with ovarian cancer from different races. M2 was validated using an internal 10-fold cross-validation only due to the lack of cancer specific site variables in SEER. All of the above was repeated for serous ovarian cancer subtype.^28^

## Results

### Data description and patient characteristics

A total of 3,510 patients from TCR who underwent treatment were retained after exclusions (Fig. 1). The SEER database consists of a total of 27,913 newly patients who diagnosed with ovarian cancer from 2009 to 2015. Similar exclusions were implemented for SEER, and a total of 3,669 White patients, 225 Blacks, and 201 Asians were included as the validation data. Table 1 gives a detailed account of the distribution of study subjects based on all clinico-pathological variables related to epithelial ovarian cancer from TCR and SEER. Majority of patients with ovarian cancer are aged between 45 and 55 years old. The age distribution in Asians from SEER was similar to that of patients from TCR and, compared with the Whites and Blacks, exhibited a younger onset. Subjects diagnosed with serous histology were the largest group, followed by endometrioid, clear cell, and mucinous for TCR, as well as for White and Black patients in SEER, while for Asians from SEER serous histology was most prevalent followed by clear cell, endometrioid, and mucinous. When patients were stratified by tumor grade, where grade 1 (well differentiated) and grade 2 (moderately differentiated) were grouped as high and grade 3 (poor differentiated) and grade 4 (undifferentiated) were grouped as low, the proportion of patients with early-stage ovarian cancer was higher among all patients (TCR, White, Black, and Asians from SEER). Proportion of patients with lymph node invasion and metastasis were lower in all study subjects from both TCR and SEER, when compared to no lymph node invasion and metastasis, while the lymph node ratio (LNR) was lower in patients from TCR as opposed to all patients from SEER (White, Black, and Asians).

**Table 1.** Descriptive Data for Demographic and Clinico-Pathological Factors for Patients with Ovarian Cancer from TCR and SEER (White, Black, and Asian)

Feature	TCR data (*n* = 3510)	White in SEER (*n* = 3669)	Black in SEER (*n* = 225)	Asian in SEER (*n* = 201)
Age at diagnosis				
18－39	488 (13.9%)	192 (5.2%)	20 (8.9%)	13 (6.5%)
40－49	1,040 (29.6%)	539 (14.7%)	41 (18.2%)	60 (29.9%)
50－59	1,198 (34.1%)	1,148 (31.3%)	68 (30.2%)	66 (32.8%)
60+	784 (22.3%)	1,790 (48.8%)	96 (42.7%)	62 (30.8%)
Histology type				
Serous	1485 (42.3%)	2,427 (66.1%)	158 (70.2%)	87 (43.3%)
Clear cell	725 (20.7%)	315 (8.6%)	14 (6.2%)	53 (26.4%)
Endometrioid	774 (22.1%)	677 (18.5%)	37 (16.4%)	49 (24.4%)
Mucinous	526 (15.0%)	250 (6.8%)	16 (7.1%)	12 (6.0%)
Tumor grade				
Low	1,442 (41.1%)	1,003 (27.3%)	51 (22.7%)	55 (27.4%)
High	2,068 (58.9%)	2,666 (72.7%)	174 (77.3%)	146 (72.6%)
Tumor size stage				
1	1571 (44.8%)	1,145 (31.2%)	53 (23.6%)	88 (43.8%)
2	479 (13.6%)	576 (15.7%)	28 (12.4%)	47 (23.4%)
3	1460 (41.6%)	1,948 (53.1%)	144 (64.0%)	66 (32.8%)
Lymph node stage				
Without	2,538 (72.3%)	2,305 (62.8%)	128 (56.9%)	141 (70.1%)
With	972 (27.7%)	1,364 (37.2%)	97 (43.1%)	60 (29.9%)
Metastasis				
Without	3183 (90.7%)	3,119 (85.0%)	189 (84.0%)	180 (89.6%)
With	327 (9.3%)	550 (15.0%)	36 (16.0%)	21 (10.4%)
Chemotherapy				
Without	496 (14.1%)	660 (18.0%)	26 (11.6%)	46 (22.9%)
With	3,014 (85.9%)	3,009 (82.0%)	199 (88.4%)	155 (77.1%)
Lymph node ratio	0.1 (0.2)	0.2 (0.3)	0.3 (0.3)	0.3 (0.3)

TCR, Taiwan Cancer Registry; SEER, Surveillance, Epidemiology, and End Results.

### Prognostic models

All clinico-pathological and demographic variables which were found to be significantly associated with CSS (Tables 2 and (3) were used to perform a stepwise regression analysis, where each interaction term was added iteratively, and finally the set of variables with the smallest AIC was used as predictors of CSS in both models M1 and M2 of this study.

**Table 2.** Hazards of Cancer-Specific Mortality in Model M1 (*n* = 3,510)

	Univariate		Multivariate	
Feature	HR (95% CI)	*p* value	Mean HR (95% CI)	*p* value
Age at diagnosis				
18－39	—	—	—	—
40－49	1.27 (0.98–1.65)	0.075	0.92 (0.62–1.39)	0.711
50－59	1.5 (1.17–1.94)	0.002	1.01 (0.68–1.48)	0.8
60+	2.29 (1.77–2.96)	<0.001	1.68 (1.14–2.5)	0.012
Histology type				
Serous	—	—	—	—
Clear cell	0.71 (0.59–0.86)	<0.001	2.14 (1.59–2.88)	<0.001
Endometrioid	0.44 (0.36–0.54)	<0.001	0.83 (0.6–1.15)	0.29
Mucinous	0.42 (0.33–0.54)	<0.001	2.35 (1.6–3.45)	<0.001
Tumor grade				
Low	—	—	—	—
High	2.39 (2.03–2.81)	<0.001	7.56 (3.58–15.97)	<0.001
Pathological T				
1	—	—	—	—
2	3.86 (2.88–5.16)	<0.001	3.86 (2.79–5.33)	<0.001
3	9.26 (7.4–11.57)	<0.001	7.49 (5.61–10.02)	<0.001
Pathological N				
Without	—	—	—	—
With	3.77 (3.27–4.35)	<0.001	1.43 (0.84–2.44)	0.218
Pathological M				
Without	—	—	—	—
With	3.74 (3.14–4.45)	<0.001	2.83 (2.08–3.86)	<0.001
Chemotherapy				
Without	—	—	—	—
With	2.47 (1.85–3.29)	<0.001	2.89 (1.47–5.7)	0.003
Lymph node ratio	6.87 (5.56–8.49)	<0.001	8.23 (2.81–24.12)	0.001
Interaction terms				
Pathological N * Pathological M	0.31 (0.22–0.45)	<0.001	0.51 (0.34–0.75)	0.001
Grade high * Chemotherapy	0.12 (0.06–0.25)	<0.001	0.14 (0.07–0.31)	<0.001
Age at diagnosis * Pathological N				
18－39 * Pathological N	—	—	—	—
40－49 * Pathological N	0.93 (0.55–1.58)	0.794	1.12 (0.63–1.97)	0.708
50－59 * Pathological N	0.76 (0.46–1.27)	0.302	1.02 (0.59–1.76)	0.816
60+ * Pathological N	0.37 (0.22–0.63)	<0.001	0.52 (0.3–0.91)	0.029
Histology type * Pathological N				
Serous * Pathological N	—	—	—	—
Clear cell * Pathological N	2.72 (1.86–3.98)	<0.001	1.37 (0.9–2.09)	0.175
Endometrioid * Pathological N	3.26 (2.14–4.98)	<0.001	2.37 (1.51–3.73)	<0.001
Mucinous * Pathological N	5.23 (3.07–8.9)	<0.001	1.99 (1.11–3.57)	0.028
Chemotherapy * Lymph node ratio	0.1 (0.05–0.22)	<0.001	0.27 (0.09–0.79)	0.028

HR, hazards ratio; Pathological M; metastasis; Pathological N, lymph node invasion; Pathological T, tumor stage.

All variables, including age over 60, histology (clear cell and mucinous), tumor grade, pathological T (tumor stage), pathological M (metastasis), chemotherapy, and the proportion of lymph node invasion, were found to be significantly associated with CSS in both univariate and multivariate analysis (Table 2). Compared to serous ovarian cancer, both clear cell and mucinous ovarian cancers demonstrated a higher risk of mortality in multivariate regression (clear cell, HR = 2.14, *p* < 0.001; mucinous, HR = 2.35, *p* < 0.001); however, there was no significant difference between the endometrioid and serous subtype. All of the TNM variables (pathological T, pathological N, pathological M) reached significance for univariate model. For the multivariate model, pathological tumor stages 2 and 3 demonstrated a risk of death 3.86 times (*p* < 0.001) and 7.49 times (*p* < 0.001) that of stage 1, and when the patient had distant metastasis (pathological M), the risk of death became 2.83 times to that of non-metastasis (*p* < 0.001). Chemotherapy was reported as a significant risk factor demonstrating a higher risk of mortality in comparison to patients with no chemotherapy (HR: 2.89, *p* = 0.003). Moreover, with one unit rise in LNR, the risk of death increases 8.23 times versus no increase in LNR (*p* = 0.001). Also, high grade of the tumor confers a higher risk of mortality (HR = 7.56, *p* < 0.001) in comparison to low grade tumors. The interaction terms that were included are provided in Table 2. In patients with poorly differentiated tumors, undergoing chemotherapy conferred a protective effect on the risk of mortality (HR = 0.14, *p* < 0.001). The risk of mortality increased in patients with histological subtypes endometrioid and mucinous, with lymph node invasion (endometrioid*pathological N, HR = 2.37, *p* < 0.001; mucinous*pathological N, HR = 1.99, *p* = 0.028). In patients undergoing chemotherapy, a greater proportion of involved lymph nodes decreased the risk of death (HR = 0.27, *p* = 0.028).

Model M2 included ovarian cancer site-specific features in addition to the clinico-pathological variables used in model M1 (Table 3). The sample size used to develop M2 was smaller (1,871 patients) for the reasons mentioned in the Methods section. Similar to model M1, most of the clinico-pathological variables were significant. Ovarian cancer site-specific biomarker CA125 after treatment and residual tumor status (with or without) after primary cytoreduction surgery were considered. Higher CA125 values and the presence of residual tumor after surgery conferred a significantly higher risk of mortality [CA125 35–100 µg/mL, HR = 2.29, *p* < 0.001; CA125 > 100 µg/mL, HR = 3.7, *p* < 0.001; residual tumor (Yes), HR = 1.43, *p* = 0.005]. Chemotherapy for advanced tumors (high grade and lymph node invasion) conferred a significant protective effect from risk of mortality due to ovarian cancer.

**Table 3.** Hazards of Cancer-Specific Mortality with Model M2

	Univariate		Multivariate	
Feature	HR (95% CI)	*p* value	HR (95% CI)	*p* value
Histology type				
Serous	—	—	—	—
Clear cell	0.79 (0.62–1)	0.054	2.88 (2.21–3.76)	<0.001
Endometrioid	0.44 (0.33–0.6)	<0.001	1.54 (1.09–2.16)	0.019
Mucinous	0.42 (0.29–0.61)	<0.001	3.61 (2.32–5.6)	<0.001
Tumor grade				
Low	—	—	—	—
High	3.08 (2.37–4.01)	<0.001	6.53 (2.45–17.4)	<0.001
Pathological T				
1	—	—	—	—
2	4.53 (2.99–6.86)	<0.001	5.07 (3.26–7.88)	<0.001
3	9.75 (7.04–13.52)	<0.001	7.88 (5.25–11.81)	<0.001
Pathological N				
Without	—	—	—	—
With	3.79 (3.1–4.62)	<0.001	7.37 (3.29–16.52)	<0.001
Pathological M				
Without	—	—	—	—
With	3.1 (2.42–3.96)	<0.001	1.54 (1.18–1.99)	0.002
Chemotherapy				
Without	—	—	—	—
With	2.13 (1.44–3.15)	<0.001	3.41 (1.29–9.03)	0.017
Cancer site-specific features				
CA125 after treatment (µg/mL)				
0–35	—	—	—	—
35–100	3.88 (2.84–5.29)	<0.001	2.29 (1.65–3.18)	<0.001
>100	7.29 (5.41–9.82)	<0.001	3.7 (2.68–5.09)	<0.001
Residual tumor status after primary cytoreduction surgery				
Without	—	—	—	—
With	3.24 (2.6–4.03)	<0.001	1.43 (1.12–1.83)	0.005
^a^Interaction terms				
Grade high * Chemotherapy	0.12 (0.04–0.36)	<0.001	0.22 (0.08–0.6)	0.004
Pathological N * Chemotherapy	0.1 (0.05–0.22)	<0.001	0.21 (0.09–0.48)	0.001

^a^ Only the interaction terms that were included in the model using the step wise hazards regression and Akaike information criterion are demonstrated.

HR, hazards ratio; Pathological M; metastasis; Pathological N, lymph node invasion; Pathological T, tumor stage.

### Model evaluation

Discrimination analysis was performed for evaluation of both M1 and M2: M1 on both TCR and SEER, and M2 only on TCR. Table 4 summarizes the results. Harrell’s c-index for TCR in M1 was 0.8, showing excellent predictive ability of the model. For all populations of SEER, the c-indices were all >0.76, again showing that M1 is an effective predictor of survival for patients of different racial origin. Furthermore, calibration analysis for 6 years, one year at a time, was performed to determine if there exists a significant difference between the number of predicted and observed deaths. The differences in the proportions were all <5% for both TCR training and testing datasets for M1, though most did not reach statistical significance (Table 5). For “overall SEER” the differences were mostly significant, but for specific races, the differences between predicted and observed events were not significant (Table 5). All findings indicated that M1 is an effective predictor of CSS. Results from SEER reiterated that M1 is also applicable to patients of different genetic ancestry. M2 also demonstrated robust performance *via* discrimination and calibration analysis (Table 4 and Supplementary Table S2). M2 had a c-index of 0.83, implying high discrimination ability for both training and testing data through internal cross-validation (Table 4). Model M2 also demonstrated good prediction performance, as the differences between predicted and observed predictions were not significantly different (*p* > 0.05) (Supplementary Table S2). Overall, all evidence indicates that both the prognostic models, M1 and M2, are effective at predicting survival in ovarian cancer patients.

**Table 4.** Harrell’s c-Index Calculated for Ovarian Cancer-Specific Survival in Different Data Sets

Dataset	*c*-index (standard error)
Model 1 (M1)	
Training	0.79 (0.009)
Testing	0.8 (0.023)
White	0.764 (0.005)
Black	0.764 (0.017)
Asian	0.762 (0.022)
Model 2 (M2)	
Training	0.81 (0.011)
Testing	0.83 (0.033)

**Table 5.** Calibration Analysis Results for Cancer-Specific Survival in Model M1 for Different Datasets

Calibration year	Mean cases	Observed	Predicted	Difference (%)	*p* value
Training					
1	3159	147	153.4	0.20	0.705
2	3012	327	347.7	0.66	0.398
3	2356	446	448.8	0.11	0.918
4	1734	452	499.5	2.28	0.079
5	1229	390	424.3	2.16	0.163
6	851	295	321.2	2.31	0.216
Testing					
1	351	16	16.4	0.11	0.947
2	335	37	37.8	0.21	0.927
3	260	45	49.1	1.39	0.645
4	200	43	56.0	5.59	0.142
5	149	42	52.2	5.47	0.225
6	108	33	40.2	5.16	0.326
SEER data (all)					
1	8110	459	502.9	0.54	0.05
2	7651	1031	1141.7	1.45	0.001
3	5943	1412	1445.3	0.56	0.381
4	4445	1492	1679.4	4.22	<0.001
5	3289	1431	1492.4	1.87	0.112
SEER White					
1	7224	408	447.2	0.54	0.064
2	6816	906	1015.9	1.61	0.001
3	5316	1257	1290.9	0.64	0.346
4	3988	1338	1510.2	4.32	<0.001
5	2963	1274	1337.3	2.13	0.084
SEER Black					
1	486	31	33	0.41	0.727
2	455	82	74.7	−1.6	0.399
3	330	103	93.7	−2.82	0.337
4	241	95	102	2.91	0.488
5	176	100	95.1	−2.76	0.619
SEER Asian					
1	400	20	22.7	0.68	0.569
2	380	43	51.1	2.13	0.257
3	297	52	60.7	2.94	0.263
4	216	59	67.2	3.78	0.319
5	150	57	60	1.97	0.702

SEER, Surveillance, Epidemiology, and End Results.

### Subtype-specific model development: serous ovarian cancer

A total of 2,184 patients with serous ovarian cancer were selected for a subtype-specific analysis. Univariate Cox proportional hazards regression analysis and stepwise regression analysis with CSS as the endpoint were conducted again to develop the serous-specific prediction model serous-M1. Supplementary Table S3 tabulates the results from the univariate and multivariate Cox proportional hazards regression analyses. Increasing age was a significant predictor of mortality in patients with serous ovarian cancer. Patients with the serous subtype were at 27 times higher risk for death if they had undifferentiated versus differentiated tumors (high vs. low grade: HR = 27.71, *p* = 0.001). Similar to all patients with epithelial ovarian cancer, those with advanced tumor stage (high TNM values) were at higher risk of mortality than those with early stages. Patients with higher LNR were at a significantly higher risk of death (HR: 11.52, *p* = 0.001). Interaction effects demonstrated that chemotherapy in serous patients with high grade tumors or in those with lymph node invasion had a protective effect.

The second model, serous-M2, was developed like before with all clinico-pathological variables and cancer site-specific variables (Supplementary Table S4). Again, patients with advanced TNM staging had higher risk in comparison to early-stage serous patients. Cancer site-specific factors, CA125 and residual tumor status, were shown to confer large and significant risks for mortality, with HRs of 14.02 (CA125 > 100 µg/mL) and 83.12 (residual tumor = yes).

### Overall survival as outcome

Prediction models for overall survival in patients with ovarian cancer were developed using TCR, and all results are demonstrated *via* Supplementary Table S5–S11. The results for the model with all clinico-pathological variables were further validated using SEER. The results were quite similar to the ones reported for ovarian CSS. The models’ performance using discrimination and calibration analysis also indicated all the models to demonstrate good performance as predictors of overall survival in patients with epithelial ovarian cancer.

## Discussion

### Principle findings

Our study analyzed patients with ovarian epithelial cancer from TCR and proposed two prognostic models for predicting risk of mortality by including carefully selected ovarian cancer-related clinico-pathological variables as well as cancer site-specific variables, collected between 2009 and 2015. The first model, M1, only included clinico-pathological and demographic variables, while model M2 additionally included cancer site-specific features as predictors, such as CA125 and residual tumor status after surgery. Cancer site-specific variables reflect the treatment situation of ovarian cancer, and were therefore included in M2 with the goal of increasing the accuracy of the ovarian cancer survival prediction model. In this study, the two survival prediction models both achieved good prediction results, and the verification using the Asian dataset of the SEER database also had good prediction results. Furthermore, no significant racial differences were found in the predictive effect.

One of the established risk factors for ovarian cancer is age, and most diagnoses occur in women who are over 50 years of age. This was confirmed in the current study. Early-stage ovarian cancer is usually associated with better prognosis in comparison to advanced-stage disease,^29^ implying that disease stage and tumor characteristics impact cancer prognosis. Findings from this study indicated that higher TNM staging was associated with a higher risk of mortality. For instance, the risk was shown to be 3–6 times higher when the tumor spread to the pelvic cavity (pathological T = 2) and the abdominal cavity (pathological T = 3), respectively. Metastasis (pathological M) also conferred a 3-fold elevated risk of mortality. Poor differentiation of tumors (*i.e.,* high grade) was also associated with a higher risk of death. Moreover, cancer site-specific feature information obtained through time of surgery or treatment course can also provide information about a person’s prognosis. For example, removal of all visible tumors during surgery leads to longer survival than when residual tumor tissue is present.^30^ Model M2, which had additional site-specific variables as predictors, indicated that the risk of death when any residual tumor remains after treatment is double that in the absence of residual tumor. Also, information on the specific subtype of ovarian cancer, grade, and the effectiveness of chemotherapy can provide additional prognostic information,^31^ and all of these were included as prognostic variables in our final models. In addition, for each model, significant interacting terms were added to improve the model precision and accuracy.

An independent analysis was conducted on the subset of patients with ovarian serous adenocarcinoma, a type of epithelial ovarian cancer that constitutes approximately 90% of all ovarian cancers, with patients >65 years of age mostly affected. The established prognostic models for serous ovarian cancer, demonstrated the coefficient of variation to be significantly different from that of all patients with ovarian cancer. This could be explained by the fact that a high degree of heterogeneity is observed among different types of ovarian cancers,^32,33^ and the factors that affect the survival or risk of mortality are varies across different subtypes. This could possibly be due to the fact that some variables that affect survival in these races may not have been included in the final model developed on TCR. For such reasons, the potential role of race in the CSS of subtypes of epithelial ovarian cancer needs to be investigated, leading to establishment of separate prediction models for different subtypes to reduce prediction errors.

### Comparison with prior work

Van Houwelingen was the first to report a prognostic index for ovarian cancer.^34^ Since then, a series of studies have reported prognostic models based on clinical characteristics to stratify patients with poor survival.^35–38^ However, patients with ovarian cancer with similar clinical characteristics also exhibit differences in prognosis, which may be due to high the molecular heterogeneity of ovarian tumors and/or different molecular genetics.^39,40^ Therefore, a comprehensive study that provides evidence for accurate prognostic models is of great importance. To date only two survival prediction studies have been conducted on patients with epithelial ovarian cancer from Taiwan. The first study recruited patients between 1979 and 2008; however, it neglected to incorporate stage and treatment information.^41^ The second study included patients that were diagnosed between 2009 and 2012, and although it had information on cancer stage and chemotherapy, it lacked details about survival.^42^ Moreover, neither of these studies conducted any internal or external validation. It has been established through several prior studies that ovarian cancer survival varies across ethnicity. For instance, Black women of African American heritage demonstrate disproportionately higher mortality by ovarian cancer in comparison to the Caucasian Americans or White women of European heritage and the phenomenon is frequently observed globally.^43^ Notably there has been a lack of inclusion of Asian and Native Hawaiian/Pacific Islander participants in prior studies and for those which reported them did that in aggregation.^44^ Only one prospective multiethnic cohort study, including 155 White patients with epithelial ovarian cancer, 93 Black, 57 Native Hawaiian, 161 Japanese American, and 141 Hispanic women was conducted, which failed to report any statistically significant ethnicity effect on risk factors. However, inverse associations were observed for parity and oral contraceptive uses among Japanese American women, while age at natural menopause and postmenopausal hormone usage were found to be associated with increased epithelial ovarian cancer risk only among Hispanic women.^44^ Overall, all Asian ethnicities have been reported with significantly lower ovarian cancer incidence rates in comparison to non-Hispanic Whites and trends have seemed to vary among different Asian subpopulations. Testing (established) European or African models on Asians is a challenge, due to the unavailability or absence of few or several predictors. Hence, studies such as the current one are important addition toward understanding and predicting prognosis of epithelial ovarian cancer in Taiwanese women thereby disaggregating the abovementioned heterogeneity of Asian population.^43,45,46^

Ovarian cancer is a fatal disease with no real effective screening^47^ and almost 70% of patients are diagnosed with it at an advanced stage, leading to poor prognosis despite aggressive and immediate treatments. Currently, there exists no test effective enough to conduct a population-specific prognostic inference for ovarian cancer. CA125 is a protein produced by ovarian cancer cells that is used as a blood marker for ovarian cancer progression and diagnosis. However, raised CA125 levels could be indicators of other conditions such as menstruation, endometriosis, or ovarian cysts, and half of early-stage ovarian cancer cases do not demonstrate elevated CA125 levels. Moreover, CA125 testing is more reliable in postmenopausal women and hence is not a recommended screening test for asymptomatic ovarian cancer cases. Also, for symptomatic cases, the CA125 test alone is not conclusive enough and is therefore coupled with transvaginal ultrasound to investigate ovarian cancer.^48,49^ Hence prognostic models like those proposed in this study can enable monitoring of women with no apparent symptoms but who are at an elevated risk of developing the disease. Human epididymis protein 4 (HE4) is another biomarker that is used for ovarian epithelial cancer. It is a protease inhibitor overexpressed especially in serous and endometrioid tumors when compared to healthy subjects.^48,50^ HE4 can be measured in the serum of women with ovarian cancer. As HE4 doesn’t demonstrate falsely elevated numbers even with benign gynecological and medical conditions and is high in the >50% of ovarian cancers that do not express CA125, HE4 has been used in along with CA125 in pre-operative assessments in women with pelvic masses.^50,51^ However, it is not a fool-proof biomarker, as many variables such as age, smoking, renal function, and non-gynecologic cancers affect HE4 serum levels.^52,53^ Therefore, population-specific prognostic models which take into consideration several clinical, demographic, and cancer site-specific factors can be successfully used in risk stratification of patients who have undergone surgery and chemotherapy. This may further allow early ovarian cancer detection, thus potentially avoiding poor prognosis in the foreseeable future.

### Limitations

One of the limitations of this study is that TCR only consists of clinico-pathological variables and therefore genetic biomarkers or genetic risk scores were not included as predictors in the final models. Several studies have shown the importance of genetic biomarkers in the prediction of ovarian CSS. However, inclusion of genetic biomarkers for risk stratification requires genetic testing which is both time-intensive and expensive. Hence, utilizing only clinico-pathological variables in clinical practice is a more feasible and quicker approach. Moreover, the models proposed in this study performed with high discrimination and precision, and therefore could be faster alternatives to genetic prediction models.

## Conclusion

Ovarian cancer is identified at an advanced stage in over two-thirds of patients, with a 5-year survival rate of only 36% and 17% for stage III and stage IV, respectively. In comparison, significantly better survival (89%) is observed in patients who are diagnosed at early stages, especially when the cancer hasn’t spread and is confined within the ovary. Prognostic models M1 and M2 can be utilized on patients who have undergone de-bulking and chemotherapy to identify the ones with higher risk of poor prognosis, early on, leading to preventive treatment and avoidance of worse outcomes through shared decision making between physicians and patients. Last but not the least, the findings from this study will be utilized to create an online platform that would allow users to predict outcomes for ovarian cancer patients after surgery using the predictors that were identified in this study.

## List of Abbreviations

AIC: Akaike information criterion
CA125: Carbohydrate Antigen 125
CSS: cancer specific survival
HE4: Human epididymis protein 4
HR: hazard ratio
LNR: lymph node ratio
M: Metastasis
M1: Model 1
M2: Model 2
N: Node
OS: overall survival
SEER: Surveillance, Epidemiology, and End Results
T: tumor stage
TCR: Taiwan Cancer Registry
