# Supplementary material for: Predicting Survival Outcomes for Patients with Ovarian Cancer Using National Cancer Registry Data from Taiwan: A Retrospective Cohort Study
Source: Womens Health Rep (New Rochelle). 2025 Jan 21;6(1):90–101. doi: 10.1089/whr.2024.0166 (PMC11773178; doi:10.1089/whr.2024.0166)
Supplement: Supplementary Data S1 [file whr.2024.0166_supplementary_data_s1.docx]

**Supplementary Materials**

**Exclusion criteria**

Two separate analyses were conduced: first, 3,510 study subjects were included to develop prognostic model 1 (M1) where 2,112 patients were excluded based on several exclusion criteria (“exclusion criteria 1”) : treatment not pursued in the reporting hospital; dead before receiving any treatment; under the age of 18 at diagnosis; diagnosis not confirmed by histology; without treatment; no tumor at the primary site; without surgery; and missing data on tumor stage, tumor grade, and lymph node status. A total of 3,210 subjects were used in a 9:1 ratio to train and test M1 via a 10-fold cross-validation strategy. For the next part, a second prognostic model (M2) was developed using additional ovarian cancer and tumor-specific variables. Here, in addition to the exclusion criteria 1, patients with missing information on CA125 and/or status and status of residual tumor after treatment were further excluded (exclusion criteria 2). On the remaining population of 1,871 patients, internal validation using 10-fold cross-validation was implemented for M2. **Figure 1** summarizes all the inclusions and exclusions and gives an overall summary of the study design. As tumor-specific variables were only collected since 2011, not all patients included in the study had this information, leading M2 to be developed using a subset of patients.

**SEER study cohort for external validation**

External validation was conducted for model M1 with subjects from different races: white, black and Asian patients from the SEER database ^19,21^. In SEER, the Asian population includes subjects of Chinese, Japanese, Korean, Vietnamese, and Laotian origin. This was done to confirm the efficiency of model M1 in predicting survival for ovarian cancer patients of Asian origin and further establish if race had a confounding effect on the prediction. Data preprocessing and exclusion criteria that were applied were the same as that of the TCR dataset. As SEER doesn’t contain cancer site-specific information, it couldn’t be utilized for validating model M2.

***Variables***

Data from the long form version of TCR was utilized in this study ^20^. It provides detailed information on each patient’s demographic data such as age, gender, height, weight, smoking, and alcohol use, as well as clinical data such as tumor histology (serous, clear-cell, endometrioid, and mucinous tumor), tumor size, pathological stage (I, II, III, IV), lymph node ratio (LNR; the proportion of excised lymph nodes positive for cancer), patient treatment methods (chemotherapy (yes, no)) and dosages, recurrence information, cancer site-specific factors (CA125 value after treatment (normal: 0-35 ug/mL, moderately high: 30-100 ug/mL, and very high: >100 ug/mL) and residual tumor status after treatment), survival status, etc. Prior studies have demonstrated that LNR ≥0.42 is a significant and independent predictor of risk of mortality in ovarian cancer patients and therefore was included as a variable under consideration for the proposed models ^22^. Ovarian cancer site-specific variables that were used to develop M2 were not available in SEER and therefore M2 was validated via cross-validation only and not by SEER.

***Study endpoints***

The primary study outcome for each of the models M1 and M2 was CSS. CSS was determined from the date of diagnosis until death due to ovarian cancer only. In addition, prognostic models for OS were developed for ovarian cancer patients following the same analysis procedure. OS was determined by following patients from the date of diagnosis of ovarian cancer to death or last follow-up, where the death is not restricted to ovarian cancer only.
