# Supplementary material for: Predicting Survival Outcomes for Patients with Ovarian Cancer Using National Cancer Registry Data from Taiwan: A Retrospective Cohort Study
Source: Womens Health Rep (New Rochelle). 2025 Jan 21;6(1):90–101. doi: 10.1089/whr.2024.0166 (PMC11773178; doi:10.1089/whr.2024.0166)
Supplement: Supplementary Table S1 [file whr.2024.0166_supplementary_table_s1.docx]

**Supplementary Tables**

**Table S1. ICD codes used for selection of study subjects**

| **Phenotype** | **ICD code (s)** |
| --- | --- |
| **Primary site:** Ovarian Cancer | C56 |
| **Morphology:** Epithelial Ovarian cancer | 3 |
| **Subtypes** |  |
| Serous ovarian cancer | 8050, 8441, 8442, 8460, 8461, 8462 |
| Mucinous ovarian cancer | 8470, 8471, 8472, 8473, 8474, 8480, 8481, 8482, 8490 |
| Endometrium ovarian cancer | 8380, 8381, 8382, 8383, 8560, 8570, 8950, 8980 |
| Bright cell ovarian cancer | 8310, 8313 |
|  |  |

ICD codes: <http://sc-dr.tw/news/104/ICD9210/ICD-9-CM_ICD-10-CM.pdf>
