# Supplementary material for: Predicting Survival Outcomes for Patients with Ovarian Cancer Using National Cancer Registry Data from Taiwan: A Retrospective Cohort Study
Source: Womens Health Rep (New Rochelle). 2025 Jan 21;6(1):90–101. doi: 10.1089/whr.2024.0166 (PMC11773178; doi:10.1089/whr.2024.0166)
Supplement: Supplementary Table S2 [file whr.2024.0166_supplementary_table_s2.docx]

**Table S2. Calibration analysis results for cancer-specific survival in model M2 for TCR data**

|  | Calibration  year | mean No. of cases | mean Observed | mean Predicted | Difference (%) | P value |
| --- | --- | --- | --- | --- | --- | --- |
| training | 1 | 1738 | 82 | 85.3 | 0.19 | 0.794 |
|  | 2 | 1656 | 178 | 192.7 | 0.84 | 0.420 |
|  | 3 | 1166 | 228 | 232.4 | 0.33 | 0.823 |
|  | 4 | 700 | 195 | 234.0 | 4.60 | 0.029 |
|  | 5 | 340 | 128 | 151.8 | 5.19 | 0.088 |
|  | 6 | 151 | 70 | 76.9 | 3.18 | 0.484 |
| testing | 1 | 194 | 11 | 7.7 | -1.70 | 0.433 |
|  | 2 | 183 | 21 | 17.4 | -1.86 | 0.541 |
|  | 3 | 142 | 27 | 24.7 | -1.40 | 0.731 |
|  | 4 | 82 | 23 | 22.7 | -0.28 | 0.962 |
|  | 5 | 45 | 17 | 16.4 | -1.01 | 0.899 |
|  | 6 | 19 | 10 | 9.1 | -2.95 | 0.811 |
