# Supplementary material for: Predicting Survival Outcomes for Patients with Ovarian Cancer Using National Cancer Registry Data from Taiwan: A Retrospective Cohort Study
Source: Womens Health Rep (New Rochelle). 2025 Jan 21;6(1):90–101. doi: 10.1089/whr.2024.0166 (PMC11773178; doi:10.1089/whr.2024.0166)
Supplement: Supplementary Table S3 [file whr.2024.0166_supplementary_table_s3.docx]

**Table S3. Hazards of cancer-specific mortality (model M1) for subtype serous ovarian cancer (N= 2,184)**

| Feature | Univariate | | Multivariate | |
| --- | --- | --- | --- | --- |
|  | **HR (95% CI)** | **P value** | **HR (95% CI)** | **P value** |
| Age at diagnosis | | | | |
| 18－39 | － | － | － | － |
| 40－49 | 1.42 (0.91-2.2) | 0.118 | 2.38 (2.38-2.38) | 0.126 |
| 50－59 | 1.62 (1.07-2.48) | 0.024 | 4.22 (4.22-4.22) | 0.007 |
| 60+ | 2.13 (1.4-3.25) | <0.001 | 5.62 (5.62-5.62) | 0.001 |
| Tumor grade | | | | |
| Low | － | － | － | － |
| High | 1.57 (1.22-2.02) | <0.001 | 27.71 (27.71-27.71) | 0.001 |
| Pathological T | | | | |
| 1 | － | － | － | － |
| 2 | 2.69 (1.54-4.72) | 0.001 | 2.21 (2.21-2.21) | 0.012 |
| 3 | 6.35 (4-10.06) | <0.001 | 4.1 (4.1-4.1) | <0.001 |
| Pathological N | | | | |
| Without | － | － | － | － |
| With | 2.2 (1.81-2.67) | <0.001 | 1.34 (1.34-1.34) | 0.058 |
| Pathological M | | | | |
| Without | － | － | － | － |
| With | 2.46 (1.97-3.07) | <0.001 | 11.64 (11.64-11.64) | 0.002 |
| Chemotherapy | | | | |
| Without | － | － | － | － |
| With | 1.59 (1.04-2.44) | 0.034 | 2.83 (2.83-2.83) | 0.194 |
| Lymph node ratio | 3.85 (2.95-5.03) | <0.001 | 11.52 (11.52-11.52) | 0.001 |
|  |  |  |  |  |
| Interaction terms |  |  |  |  |
| Age at diagnosis * Lymph node ratio | | | | |
| 18－39 *  Lymph node ratio | － | － | － | － |
| 40－49 *  Lymph node ratio | 0.69 (0.22-2.22) | 0.536 | 1.1 (1.1-1.1) | 0.738 |
| 50－59 *  Lymph node ratio | 0.39 (0.12-1.24) | 0.111 | 0.51 (0.51-0.51) | 0.351 |
| 60+ *  Lymph node ratio | 0.16 (0.05-0.51) | 0.002 | 0.25 (0.25-0.25) | 0.069 |
|  |  |  |  |  |
| Pathological N *  Pathological M | 0.47 (0.29-0.77) | 0.002 | 0.54 (0.54-0.54) | 0.023 |
|  |  |  |  |  |
| Chemotherapy * Tumor grade high | 0.21 (0.07-0.65) | 0.007 | 0.14 (0.14-0.14) | 0.014 |
|  |  |  |  |  |
| Chemotherapy * Pathological M | 0.06 (0.02-0.2) | <0.001 | 0.23 (0.23-0.23) | 0.064 |
|  |  |  |  |  |
| Tumor grade high * Lymph node ratio | 0.23 (0.11-0.46) | <0.001 | 0.3 (0.3-0.3) | 0.009 |
|  | | | | |
| Age at diagnosis * Tumor grade | | | | |
|  | | | | |
| 18－39 *  Tumor grade high | － | － | － | － |
| 40－49 *  Tumor grade high | 0.45 (0.16-1.22) | 0.115 | 0.32 (0.32-0.32) | 0.052 |
| 50－59 *  Tumor grade high | 0.34 (0.13-0.88) | 0.027 | 0.26 (0.26-0.26) | 0.018 |
| 60+ *  Tumor grade high | 0.32 (0.13-0.82) | 0.017 | 0.27 (0.27-0.27) | 0.02 |

Pathological T: Tumor stage, Pathological N: Lymph node invasion, Pathological M: Metastasis, HR: Hazards Ratio
