# Supplementary material for: Predicting Survival Outcomes for Patients with Ovarian Cancer Using National Cancer Registry Data from Taiwan: A Retrospective Cohort Study
Source: Womens Health Rep (New Rochelle). 2025 Jan 21;6(1):90–101. doi: 10.1089/whr.2024.0166 (PMC11773178; doi:10.1089/whr.2024.0166)
Supplement: Supplementary Table S4 [file whr.2024.0166_supplementary_table_s4.docx]

**Table S4. Hazards of cancer specific mortality (model M2) for subtype serous ovarian cancer**

| **Feature** | **Univariate** | | **Multivariate** | |
| --- | --- | --- | --- | --- |
|  | **HR (95% CI)** | **P value** | **HR (95% CI)** | **P value** |
| **Age at diagnosis** | | | | |
| 18－39 | － | － | － | － |
| 40－49 | 1.97 (0.97-4) | 0.061 | 3.09 (1.32-7.26) | 0.018 |
| 50－59 | 2.42 (1.22-4.79) | 0.011 | 4.76 (2.22-10.24) | <0.001 |
| 60+ | 2.67 (1.34-5.31) | 0.005 | 1.3 (0.9-1.88) | 0.177 |
| **Tumor grade** | | | | |
| Low | － | － | － | － |
| High | 2.48 (1.55-3.97) | <0.001 | 54.36 (6.61-447.16) | 0.009 |
| **Pathological T** | | | | |
| 1 | － | － | － | － |
| 2 | 3.39 (1.49-7.69) | 0.004 | 1.63 (1.18-2.26) | 0.007 |
| 3 | 7.67 (3.78-15.53) | <0.001 | 27.93 (3.83-203.91) | 0.029 |
| **Pathological N** | | | | |
| Without | － | － | － | － |
| With | 2.2 (1.69-2.87) | <0.001 | 1.1 (0.21-5.78) | 0.801 |
| **Pathological M** | | | | |
| Without | － | － | － | － |
| With | 2.29 (1.71-3.08) | <0.001 | 7.59 (0.92-62.33) | 0.081 |
| **CA125 lab value after treatment (µg/mL)** | | | | |
| 0-35 | － | － | － | － |
| 35-100 | 2.54 (1.69-3.8) | <0.001 | 1.18 (0.82-1.68) | 0.394 |
| >100 | 5.33 (3.67-7.74) | <0.001 | 14.02 (1.47-134.14) | 0.165 |
| **Residual tumor status after primary cytoreduction surgery** | | | | |
| Without | － | － | － | － |
| With | 2.08 (1.53-2.82) | <0.001 | 83.12 (3.56-1940.8) | 0.015 |
| **Lymph node ratio** | 3.4 (2.37-4.86) | <0.001 | 37.22 (3.99-346.79) | 0.03 |
|  |  |  |  |  |
| **Interaction terms** | | | | |
| Age at diagnosis * Lymph node ratio | | | | |
| 18－39 *  Lymph node ratio | － | － | － | － |
| 40－49 *  Lymph node ratio | 0.02 (0-0.38) | 0.009 | 4.9 (0.72-33.59) | 0.121 |
| 50－59 *  Lymph node ratio | 0.01 (0-0.19) | 0.002 | 1.13 (0.11-11.37) | 0.768 |
| 60+ *  Lymph node ratio | 0.01 (0-0.12) | 0.001 | 2.2 (0.37-13.02) | 0.38 |
| Age at diagnosis * Tumor grade | | | | |
| 18－39 *  Tumor grade high | － | － | － | － |
| 40－49 *  Tumor grade high | 0.17 (0.02-1.74) | 0.136 | 0.48 (0.05-4.41) | 0.528 |
| 50－59 *  Tumor grade high | 0.15 (0.02-1.54) | 0.111 | 1.17 (0.18-7.54) | 0.832 |
| 60+ *  Tumor grade high | 0.12 (0.01-1.02) | 0.052 | NA | NA |
| Age at diagnosis * CA125 lab value after treatment | | | | |
| 18－39 * 35-100 | － | － | － | － |
| 40－49 * 35-100 | 1.71 (0.28-10.58) | 0.561 | 0.08 (0.01-0.74) | 0.192 |
| 50－59 * 35-100 | 0.9 (0.16-4.98) | 0.901 | 0.04 (0-0.37) | 0.067 |
| 60+ * 35-100 | 0.45 (0.08-2.66) | 0.378 | 0.04 (0-0.3) | 0.041 |
| 40－49 * 100+ | 0.08 (0.01-0.74) | 0.026 | 0.02 (0-0.53) | 0.035 |
| 50－59 * 100+ | 0.02 (0-0.2) | 0.001 | 0.02 (0-0.4) | 0.022 |
| 60+ * 100+ | 0.02 (0-0.16) | <0.001 | 0.01 (0-0.21) | 0.01 |

Pathological T: Tumor stage, Pathological N: Lymph node invasion, Pathological M: Metastasis, HR: Hazards Ratio
