# Supplementary material for: Predicting Survival Outcomes for Patients with Ovarian Cancer Using National Cancer Registry Data from Taiwan: A Retrospective Cohort Study
Source: Womens Health Rep (New Rochelle). 2025 Jan 21;6(1):90–101. doi: 10.1089/whr.2024.0166 (PMC11773178; doi:10.1089/whr.2024.0166)
Supplement: Supplementary Table S5 [file whr.2024.0166_supplementary_table_s5.docx]

**Table S5. Cox proportional hazards model M1 for overall survival**

| Feature | Univariate | | Multivariate | | |
| --- | --- | --- | --- | --- | --- |
|  | HR (95% CI) | P value | Mean HR (95% CI) | Mean P value | |
| Age at diagnosis | | | | |  |
| 18－39 | － | － | － | － | |
| 40－49 | 1.46 (1.16-1.84) | 0.001 | 1.48 (0.99-2.23) | 0.07 | |
| 50－59 | 1.6 (1.28-2.01) | <0.001 | 1.53 (1.02-2.3) | 0.054 | |
| 60+ | 2.71 (2.16-3.4) | <0.001 | 2.59 (1.74-3.87) | <0.001 | |
| Histology type | | | | |  |
| Serous | － | － | － | － | |
| Clear cell | 0.71 (0.6-0.83) | <0.001 | 1.85 (1.44-2.37) | <0.001 | |
| Endometrioid | 0.46 (0.39-0.55) | <0.001 | 0.9 (0.69-1.16) | 0.438 | |
| Mucinous | 0.42 (0.34-0.51) | <0.001 | 1.63 (1.18-2.25) | 0.004 | |
| Tumor grade | | | | |  |
| Low | － | － | － | － | |
| High | 2.21 (1.93-2.53) | <0.001 | 2.12 (1.36-3.3) | 0.002 | |
| Pathological T | | | | |  |
| 1 | － | － | － | － | |
| 2 | 2.75 (2.17-3.47) | <0.001 | 3.06 (2.35-4) | <0.001 | |
| 3 | 6.96 (5.87-8.24) | <0.001 | 6.18 (4.9-7.81) | <0.001 | |
| Pathological N | | | | |  |
| Without | － | － | － | － | |
| With | 3.47 (3.08-3.92) | <0.001 | 1.11 (0.89-1.39) | 0.375 | |
| Pathological M | | | | |  |
| Without | － | － | － | － | |
| With | 3.6 (3.1-4.19) | <0.001 | 3.25 (2.18-4.85) | <0.001 | |
| Chemotherapy | | | | |  |
| Without | － | － | － | － | |
| With | 2.17 (1.73-2.73) | <0.001 | 0.69 (0.52-0.91) | 0.027 | |
| Lymph node ratio | 6.71 (5.6-8.05) | <0.001 | 2.34 (1.75-3.12) | <0.001 | |
| Interactions terms | | | | |  |
| Age at diagnosis * Tumor grade | | | | |  |
| 18－39 *  grade high | － | － | － | － | |
| 40－49 *  grade high | 0.68 (0.42-1.11) | 0.123 | 0.65 (0.39-1.08) | 0.116 | |
| 50－59 *  Grade high | 0.54 (0.33-0.87) | 0.011 | 0.59 (0.35-0.98) | 0.052 | |
| 60+ *  Grade high | 0.39 (0.24-0.63) | <0.001 | 0.41 (0.25-0.68) | 0.001 | |
| Histology type * Pathological N | | | | | |
| Serous * Pathological N | － | － | － | － | |
| Clear cell * Pathological N | 2.6 (1.88-3.6) | <0.001 | 1.51 (1.05-2.16) | 0.039 | |
| Endometrioid * Pathological N | 2.54 (1.78-3.63) | <0.001 | 1.88 (1.28-2.76) | 0.003 | |
| Mucinous * Pathological N | 4.13 (2.56-6.65) | <0.001 | 2.23 (1.31-3.8) | 0.005 | |
| Grade high * Pathological M | 0.41 (0.29-0.6) | <0.001 | 0.7 (0.47-1.05) | 0.087 | |
| Pathological N * Pathological M | 0.4 (0.29-0.54) | <0.001 | 0.6 (0.42-0.84) | 0.007 | |
