# Supplementary material for: Predicting Survival Outcomes for Patients with Ovarian Cancer Using National Cancer Registry Data from Taiwan: A Retrospective Cohort Study
Source: Womens Health Rep (New Rochelle). 2025 Jan 21;6(1):90–101. doi: 10.1089/whr.2024.0166 (PMC11773178; doi:10.1089/whr.2024.0166)
Supplement: Supplementary Table S6 [file whr.2024.0166_supplementary_table_s6.docx]

**Table S6. Results of Cox proportional hazards regression using model M2 for overall survival**

| Feature | Univariate | | Multivariate | |  |
| --- | --- | --- | --- | --- | --- |
|  | HR (95% CI) | P value | HR (95% CI) | P value |  |
| Age at diagnosis | | | | | |
| 18－39 | － | － | － | － |  |
| 40－49 | 1.62 (1.13-2.32) | 0.009 | 1.19 (0.72-1.99) | 0.514 |  |
| 50－59 | 1.66 (1.16-2.36) | 0.005 | 1.01 (0.61-1.65) | 0.78 |  |
| 60+ | 2.7 (1.9-3.84) | <0.001 | 1.96 (1.2-3.2) | 0.009 |  |
| Histology type | | | | | |
| Serous | － | － | － | － |  |
| Clear cell | 0.82 (0.66-1.02) | 0.069 | 2.93 (2.29-3.73) | <0.001 |  |
| Endometrioid | 0.51 (0.4-0.67) | <0.001 | 1.68 (1.24-2.26) | 0.002 |  |
| Mucinous | 0.45 (0.33-0.63) | <0.001 | 3.32 (2.21-5.01) | <0.001 |  |
| Tumor grade | | | | | |
| Low | － | － | － | － |  |
| High | 2.66 (2.14-3.32) | <0.001 | 2.05 (1.31-3.2) | 0.003 |  |
| Pathological T | | | | | |
| 1 | － | － | － | － |  |
| 2 | 3.59 (2.5-5.17) | <0.001 | 3.67 (2.47-5.47) | <0.001 |  |
| 3 | 8.46 (6.44-11.1) | <0.001 | 6.4 (4.5-9.09) | <0.001 |  |
| Pathological N | | | | | |
| Without | － | － | － | － |  |
| With | 3.53 (2.96-4.2) | <0.001 | 1.74 (0.86-3.5) | 0.136 |  |
| Pathological M | | | | | |
| Without | － | － | － | － |  |
| With | 3.39 (2.75-4.19) | <0.001 | 1.63 (1.3-2.05) | <0.001 |  |
| CA125 lab value after treatment (µg/mL) | | | | | |
| 0-35 | － | － | － | － |  |
| 35-100 | 3.59 (2.72-4.75) | <0.001 | 2.31 (1.71-3.11) | <0.001 |  |
| 100+ | 7.75 (5.99-10.01) | <0.001 | 3.92 (2.96-5.19) | <0.001 |  |
| Residual tumor status after primary cytoreduction surgery | | | | | |
| Without | － | － | － | － |  |
| With | 2.92 (2.42-3.52) | <0.001 | 2 (1.26-3.18) | 0.005 |  |
| Interaction terms | | | | |  |
| Age at diagnosis * Pathological N | | | | |  |
| 18－39 *  Pathological N | － | － | － | － |  |
| 40－49 *  Pathological N | 1.2 (0.58-2.51) | 0.625 | 1.17 (0.53-2.54) | 0.712 |  |
| 50－59 *  Pathological N | 1.05 (0.51-2.15) | 0.899 | 1.18 (0.55-2.54) | 0.68 |  |
| 60+ * Pathological N | 0.5 (0.24-1.02) | 0.058 | 0.58 (0.27-1.25) | 0.177 |  |
| Grade high *  with residual tumor | 0.48 (0.3-0.76) | 0.002 | 0.59 (0.35-0.97) | 0.052 |  |
