# Supplementary material for: Predicting Survival Outcomes for Patients with Ovarian Cancer Using National Cancer Registry Data from Taiwan: A Retrospective Cohort Study
Source: Womens Health Rep (New Rochelle). 2025 Jan 21;6(1):90–101. doi: 10.1089/whr.2024.0166 (PMC11773178; doi:10.1089/whr.2024.0166)
Supplement: Supplementary Table S7 [file whr.2024.0166_supplementary_table_s7.docx]

**Table S7. Harrell’s c-index calculated for overall survival with different data sets**

|  | Datasets | C-index (insert label) |
| --- | --- | --- |
| Model 1 | Training | 0.76 (0.008) |
|  | Testing | 0.78 (0.022) |
|  | White | 0.754 (0.005) |
|  | Black | 0.744 (0.017) |
|  | Asian | 0.752 (0.021) |
| Model 2 | Training | 0.80 (0.010) |
|  | Testing | 0.82 (0.029) |
