# Supplementary material for: Predicting Survival Outcomes for Patients with Ovarian Cancer Using National Cancer Registry Data from Taiwan: A Retrospective Cohort Study
Source: Womens Health Rep (New Rochelle). 2025 Jan 21;6(1):90–101. doi: 10.1089/whr.2024.0166 (PMC11773178; doi:10.1089/whr.2024.0166)
Supplement: Supplementary Table S8 [file whr.2024.0166_supplementary_table_s8.docx]

**Table S8. Calibration analysis for model M1 with overall survival as outcome**

|  | Calibration  year | mean No. of cases | mean Observed | mean Predicted | Difference | P value |
| --- | --- | --- | --- | --- | --- | --- |
| training | 1 | 3158 | 193 | 194.4 | 0.04 | 0.943 |
|  | 2 | 2965 | 453 | 454.9 | 0.06 | 0.947 |
|  | 3 | 2263 | 583 | 577.1 | -0.22 | 0.846 |
|  | 4 | 1617 | 581 | 577.4 | -0.17 | 0.901 |
|  | 5 | 1130 | 525 | 506.9 | -1.14 | 0.493 |
|  | 6 | 763 | 433 | 414.9 | -1.58 | 0.433 |
| testing | 1 | 352 | 24 | 23.2 | -0.23 | 0.901 |
|  | 2 | 328 | 51 | 54.1 | 0.87 | 0.747 |
|  | 3 | 259 | 61 | 68.2 | 2.38 | 0.477 |
|  | 4 | 194 | 60 | 69.8 | 4.03 | 0.316 |
|  | 5 | 131 | 57 | 57.9 | 0.50 | 0.920 |
|  | 6 | 86 | 45 | 46.1 | 0.91 | 0.880 |
| SEER data | 1 | 8110 | 561 | 677.7 | 1.44 | <0.001 |
|  | 2 | 7549 | 1208 | 1549.6 | 4.53 | <0.001 |
|  | 3 | 5796 | 1638 | 1925 | 4.95 | <0.001 |
|  | 4 | 4261 | 1719 | 1982.4 | 6.18 | <0.001 |
|  | 5 | 3110 | 1649 | 1786.4 | 4.42 | 0.001 |
| White | 1 | 7224 | 498 | 603.7 | 1.46 | <0.001 |
|  | 2 | 6726 | 1060 | 1381.1 | 4.77 | <0.001 |
|  | 3 | 5189 | 1456 | 1721 | 5.11 | <0.001 |
|  | 4 | 3823 | 1539 | 1783.8 | 6.4 | <0.001 |
|  | 5 | 2805 | 1469 | 1602.3 | 4.75 | 0.001 |
| Black | 1 | 486 | 41 | 43.8 | 0.57 | 0.675 |
|  | 2 | 445 | 98 | 99.9 | 0.42 | 0.852 |
|  | 3 | 317 | 122 | 123.1 | 0.34 | 0.923 |
|  | 4 | 229 | 112 | 119 | 3.06 | 0.521 |
|  | 5 | 162 | 115 | 112.1 | -1.8 | 0.782 |
| Asian | 1 | 400 | 22 | 30.2 | 2.06 | 0.134 |
|  | 2 | 378 | 50 | 68.7 | 4.94 | 0.024 |
|  | 3 | 290 | 60 | 81 | 7.23 | 0.02 |
|  | 4 | 209 | 68 | 79.7 | 5.58 | 0.192 |
|  | 5 | 143 | 65 | 72.1 | 4.94 | 0.405 |
