# Supplementary material for: Predicting Survival Outcomes for Patients with Ovarian Cancer Using National Cancer Registry Data from Taiwan: A Retrospective Cohort Study
Source: Womens Health Rep (New Rochelle). 2025 Jan 21;6(1):90–101. doi: 10.1089/whr.2024.0166 (PMC11773178; doi:10.1089/whr.2024.0166)
Supplement: Supplementary Table S9 [file whr.2024.0166_supplementary_table_s9.docx]

**Table S9. Cox proportional hazards model M1 for overall survival in serous ovarian cancer patients**

| Feature | Univariate | | Multivariate | |
| --- | --- | --- | --- | --- |
|  | *HR (95% CI)* | *P value* | *Mean HR (95% CI)* | *Mean P value* |
| Age at diagnosis | | | | |
| 18－39 | *－* | *－* | *－* | *－* |
| 40－49 | *1.65 (1.13-2.42)* | *0.01* | *3.15 (3.15-3.15)* | *0.022* |
| 50－59 | *1.64 (1.13-2.39)* | *0.009* | *4.32 (4.32-4.32)* | *0.002* |
| 60+ | *2.45 (1.69-3.54)* | *<0.001* | *6.12 (6.12-6.12)* | *<0.001* |
| Tumor grade | | | | |
| Low | *－* | *－* | *－* | *－* |
| High | *1.48 (1.21-1.83)* | *<0.001* | *53.95 (53.95-53.95)* | *<0.001* |
| Pathological T | | | | |
| 1 | *－* | *－* | *－* | *－* |
| 2 | *2.09 (1.32-3.32)* | *0.002* | *1.73 (1.73-1.73)* | *0.038* |
| 3 | *5.48 (3.81-7.87)* | *<0.001* | *3.47 (3.47-3.47)* | *<0.001* |
| Pathological N | | | | |
| Without | *－* | *－* | *－* | *－* |
| With | *2.17 (1.84-2.56)* | *<0.001* | *3.24 (3.24-3.24)* | *0.013* |
| Pathological M | | | | |
| Without | *－* | *－* | *－* | *－* |
| With | *2.44 (2.01-2.95)* | *<0.001* | *19.87 (19.87-19.87)* | *<0.001* |
| Chemotherapy | | | | |
| Without | *－* | *－* | *－* | *－* |
| With | *1.67 (1.15-2.43)* | *0.007* | *10.91 (10.91-10.91)* | *0.004* |
| Lymph node ratio | *4.06 (3.23-5.1)* | *<0.001* | *4.32 (4.32-4.32)* | *0.013* |
| Interaction terms | | | | |
| Age at diagnosis * Lymph node ratio | | | | |
| 18－39 *  Lymph node ratio | *－* | *－* | *－* | *－* |
| 40－49 *  Lymph node ratio | *0.72 (0.26-2.03)* | *0.537* | *0.75 (0.75-0.75)* | *0.666* |
| 50－59 *  Lymph node ratio | *0.58 (0.21-1.64)* | *0.306* | *0.63 (0.63-0.63)* | *0.494* |
| 60+ *  Lymph node ratio | *0.24 (0.08-0.66)* | *0.006* | *0.31 (0.31-0.31)* | *0.063* |
| Chemotherapy * grade high | *0.14 (0.05-0.42)* | *<0.001* | *0.06 (0.06-0.06)* | *<0.001* |
| Pathological M * Chemotherapy | *0.05 (0.02-0.14)* | *<0.001* | *0.08 (0.08-0.08)* | *<0.001* |
| Age at diagnosis * Tumor grade | | | | |
| 18－39 *  Tumor grade high | *－* | *－* | *－* | *－* |
| 40－49 *  Tumor grade high | *0.37 (0.15-0.9)* | *0.028* | *0.3 (0.3-0.3)* | *0.03* |
| 50－59 *  Tumor grade high | *0.26 (0.11-0.6)* | *0.002* | *0.23 (0.23-0.23)* | *0.003* |
| 60+ *  Tumor grade high | *0.25 (0.11-0.59)* | *0.001* | *0.26 (0.26-0.26)* | *0.007* |
| Chemotherapy *  Pathological N | *0.27 (0.13-0.57)* | *0.001* | *0.34 (0.34-0.34)* | *0.023* |
