# Supplementary material for: Predicting Survival Outcomes for Patients with Ovarian Cancer Using National Cancer Registry Data from Taiwan: A Retrospective Cohort Study
Source: Womens Health Rep (New Rochelle). 2025 Jan 21;6(1):90–101. doi: 10.1089/whr.2024.0166 (PMC11773178; doi:10.1089/whr.2024.0166)
Supplement: Supplementary Table S10 [file whr.2024.0166_supplementary_table_s10.docx]

**Table S10. Cox proportional hazards model M2 for overall survival in serous ovarian cancer patients**

| Feature | Univariate | | | Multivariate | |
| --- | --- | --- | --- | --- | --- |
|  | *HR (95% CI)* | *P value* | *Mean HR (95% CI)* | | *Mean P value* |
| Age at diagnosis | | | | | |
| 18－39 | *－* | *－* | | *－* | *－* |
| 40－49 | *2.34 (1.16-4.72)* | *0.017* | | *38.3 (0-Inf)* | *0.171* |
| 50－59 | *2.47 (1.25-4.89)* | *0.01* | | *58.18 (0-Inf)* | *0.135* |
| 60+ | *3.34 (1.69-6.59)* | *0.001* | | *136.72 (0-Inf)* | *0.102* |
| Tumor grade | | | | | |
| Low | *－* | *－* | | *－* | *－* |
| High | *2.04 (1.36-3.05)* | *0.001* | | *50.11 (0-Inf)* | *0.123* |
| Pathological T | | | | | |
| 1 | *－* | *－* | | *－* | *－* |
| 2 | *2.36 (1.04-5.34)* | *0.039* | | *1.88 (0.77-4.58)* | *0.188* |
| 3 | *7.67 (3.94-14.92)* | *<0.001* | | *4.89 (2.31-10.35)* | *<0.001* |
| Pathological N | | | | | |
| Without | *－* | *－* | | *－* | *－* |
| With | *2.29 (1.78-2.93)* | *<0.001* | | *1.28 (0.9-1.81)* | *0.24* |
| Pathological M | | | | | |
| Without | *－* | *－* | | *－* | *－* |
| With | *2.49 (1.91-3.26)* | *<0.001* | | *2.94 (1.54-5.64)* | *0.002* |
| CA125 lab value after treatment (µg/mL) | | | | | |
| 0-35 | *－* | *－* | | *－* | *－* |
| 35-100 | *2.34 (1.6-3.43)* | *<0.001* | | *2.05 (0.44-9.53)* | *0.369* |
| 100+ | *5.12 (3.63-7.22)* | *<0.001* | | *8.96 (0.79-102.02)* | *0.084* |
| Residual tumor status after primary cytoreduction surgery | | | | | |
| Without | *－* | *－* | | *－* | *－* |
| With | *2.09 (1.57-2.78)* | *<0.001* | | *1.19 (0.83-1.71)* | *0.376* |
| Lymph node ratio | *3.67 (2.65-5.1)* | *<0.001* | | *31.56*  *(0.75-1328.49)* | *0.086* |
| Interaction terms | | | | | |
| Age at diagnosis * Lymph node ratio | | | | | |
| 18－39 *  Lymph node ratio | *－* | *－* | | *－* | *－* |
| 40－49 *  Lymph node ratio | *0.02 (0-0.43)* | *0.011* | | *0.06 (0-2.59)* | *0.156* |
| 50－59 *  Lymph node ratio | *0.02 (0-0.28)* | *0.005* | | *0.06 (0-2.55)* | *0.154* |
| 60+ *  Lymph node ratio | *0.01 (0-0.14)* | *0.001* | | *0.02 (0-0.95)* | *0.068* |
| Age at diagnosis * Tumor grade | | | | | |
| 18－39 *  Tumor grade high | *－* | *－* | | *－* | *－* |
| 40－49 *  Tumor grade high | *0.13 (0.01-1.2)* | *0.071* | | *0.03 (0-Inf)* | *0.194* |
| 50－59 *  Tumor grade high | *0.16 (0.02-1.6)* | *0.119* | | *0.02 (0-Inf)* | *0.154* |
| 60+ *  Tumor grade high | *0.09 (0.01-0.81)* | *0.032* | | *0.02 (0-Inf)* | *0.12* |
| Age at diagnosis * CA125 lab value after treatment | | | | | |
| 18－39 * 35-100 | *－* | *－* | | *－* | *－* |
| 40－49 * 35-100 | *0.7 (0.13-3.69)* | *0.67* | | *1.75 (0.29-10.68)* | *0.558* |
| 50－59 * 35-100 | *0.49 (0.1-2.31)* | *0.369* | | *1.12 (0.21-5.89)* | *0.848* |
| 60+ * 35-100 | *0.22 (0.04-1.07)* | *0.061* | | *0.56 (0.1-3.13)* | *0.523* |
| 40－49 * 100+ | *0.07 (0.01-0.67)* | *0.021* | | *0.73 (0.05-9.7)* | *0.652* |
| 50－59 * 100+ | *0.02 (0-0.22)* | *0.001* | | *0.43 (0.03-5.43)* | *0.494* |
| 60+ * 100+ | *0.02 (0-0.15)* | *<0.001* | | *NA* | *NA* |
| Pathological M * Residual Tumor | *0.39 (0.2-0.76)* | *0.006* | | *0.51 (0.25-1.06)* | *0.092* |
