# Supplementary material for: Predicting Survival Outcomes for Patients with Ovarian Cancer Using National Cancer Registry Data from Taiwan: A Retrospective Cohort Study
Source: Womens Health Rep (New Rochelle). 2025 Jan 21;6(1):90–101. doi: 10.1089/whr.2024.0166 (PMC11773178; doi:10.1089/whr.2024.0166)
Supplement: Supplementary Table S11 [file whr.2024.0166_supplementary_table_s11.docx]

**Table S11. Calibration analysis for model M1 for serous ovarian cancer**

|  | *Calibration*  *year* | *mean No. of*  *cases (SE)* | *mean Observed*  *(SE)* | *mean Predicted*  *(SE)* | *Difference (%)* | *P value* |
| --- | --- | --- | --- | --- | --- | --- |
| *training* | 1 | 1336.5 ( 0.5 ) | 71.1 ( 1.8 ) | 72.5 ( 1.5 ) | 0.1 | 0.871 |
|  | 2 | 1265.4 ( 2.1 ) | 194.4 ( 3.5 ) | 197.5 ( 3.3 ) | 0.25 | 0.825 |
|  | 3 | 957.6 ( 7 ) | 296.1 ( 4.9 ) | 291.4 ( 3.7 ) | -0.49 | 0.784 |
|  | 4 | 638.1 ( 6.1 ) | 317.7 ( 5.8 ) | 316.5 ( 4.6 ) | -0.19 | 0.945 |
|  | 5 | 422.1 ( 6 ) | 298.8 ( 5.4 ) | 289.1 ( 4.8 ) | -2.29 | 0.569 |
|  | 6 | 278.1 ( 5 ) | 256.5 ( 6.2 ) | 248 ( 5.4 ) | -3.05 | 0.59 |
| *testing* | 1 | 148.5 ( 0.5 ) | 7.9 ( 1.8 ) | 8.1 ( 0.9 ) | 0.13 | 0.944 |
|  | 2 | 140.6 ( 2.1 ) | 21.6 ( 3.5 ) | 21.9 ( 1.2 ) | 0.25 | 0.941 |
|  | 3 | 106.4 ( 7 ) | 32.9 ( 4.9 ) | 32.4 ( 2 ) | -0.5 | 0.925 |
|  | 4 | 70.9 ( 6.1 ) | 35.3 ( 5.8 ) | 35.1 ( 2.7 ) | -0.27 | 0.974 |
|  | 5 | 46.9 ( 6 ) | 33.2 ( 5.4 ) | 32 ( 3 ) | -2.48 | 0.837 |
|  | 6 | 30.9 ( 5 ) | 28.5 ( 6.2 ) | 27.5 ( 3.4 ) | -3.39 | 0.842 |
| *SEER data* | 1 | 2672 | 166 | 249.3 | 3.12 | <0.001 |
|  | 2 | 2506 | 427 | 609 | 7.26 | <0.001 |
|  | 3 | 1834 | 608 | 822.7 | 11.71 | <0.001 |
|  | 4 | 1266 | 631 | 843.6 | 16.79 | <0.001 |
|  | 5 | 847 | 559 | 721.4 | 19.17 | <0.001 |
| *White* | 1 | 2427 | 153 | 226 | 3.01 | <0.001 |
|  | 2 | 2274 | 388 | 552.6 | 7.24 | <0.001 |
|  | 3 | 1671 | 558 | 751.6 | 11.58 | <0.001 |
|  | 4 | 1149 | 581 | 772.9 | 16.7 | <0.001 |
|  | 5 | 775 | 508 | 659.7 | 19.58 | <0.001 |
| *Black* | 1 | 158 | 8 | 15.1 | 4.52 | 0.067 |
|  | 2 | 150 | 28 | 36.4 | 5.58 | 0.165 |
|  | 3 | 103 | 36 | 46.5 | 10.18 | 0.124 |
|  | 4 | 71 | 33 | 43.5 | 14.78 | 0.112 |
|  | 5 | 41 | 37 | 37.6 | 1.56 | 0.917 |
| *Asian* | 1 | 87 | 5 | 8.2 | 3.64 | 0.268 |
|  | 2 | 82 | 11 | 20 | 11.02 | 0.044 |
|  | 3 | 60 | 14 | 24.6 | 17.74 | 0.032 |
|  | 4 | 46 | 17 | 27.2 | 22.2 | 0.05 |
|  | 5 | 31 | 14 | 24 | 32.18 | 0.042 |
